# The impact of conventional and electronic cigarette exposure on atherosclerosis development in *Rattus norvegicus*

**DOI:** 10.1186/s43044-025-00626-2

**Published:** 2025-03-26

**Authors:** I Gde Rurus Suryawan, Meity Ardiana, Tony Santoso Putra, A’rofah Nurlina Puspitasari, Priangga Adi, Wynne Widiarti, Pandit Bagus Tri Saputra

**Affiliations:** 1 Department of Cardiology and Vascular Medicine, Faculty of Medicine, Universitas Airlangga, Surabaya, Indonesia; 2 Department of Cardiology and Vascular Medicine, Dr. Soetomo General Academic Hospital, Surabaya, Indonesia; 3 Department of Anatomical Pathology, Faculty of Medicine, Universitas Airlangga, Surabaya, Indonesia; 4 Department of Anatomical Pathology, Dr. Soetomo General Academic Hospital, Surabaya, Indonesia; 5 Faculty of Medicine, Universitas Airlangga, Surabaya, Indonesia

**Keywords:** *Rattus norvegicus*, Aorta, Iliac arteries, Atherosclerosis

## Abstract

**Background:**

Smoking, including conventional and electronic cigarettes (e-cigarettes), is a major contributor to cardiovascular morbidity and mortality. Indonesia, with 69.1 million smokers, experiences a high burden of smoking-related diseases. This study aims to evaluate the impact of conventional and e-cigarette exposure on atherosclerosis in *Rattus norvegicus* (Wistar rats).

**Methods:**

Twenty-one male Wistar rats were randomized into three groups: control, conventional cigarette exposure, and e-cigarette exposure. Both smoking groups received equivalent nicotine doses for 30 min daily, five days a week, for 12 weeks. Aortic and iliac artery intima-media thickness (IMT) was measured, and plasma levels of tumor necrosis factor-alpha (TNF-α) and monocyte chemoattractant protein-1 (MCP-1) were analyzed using ELISA. Histopathological changes were also examined.

**Results:**

Cigarette exposure significantly increased IMT in the aorta (control: 67.22 ± 3.07 µm; conventional: 100.89 ± 25.60 µm; e-cigarette: 83.75 ± 7.45 µm; *p* < 0.05) and iliac arteries (control: 68.50 ± 5.6 µm; conventional: 90.49 ± 25.02 µm; e-cigarette: 90.68 ± 12.26 µm; *p* = 0.031). MCP-1 levels were significantly elevated in the conventional group (205.77 ± 22.18 pg/mL; *p* = 0.003), while TNF-α levels increased in both groups but without statistical significance. Histopathology revealed fatty streaks and elastic fiber disruption in both exposure groups, with no significant differences observed (*p* > 0.05).

**Conclusions:**

Both conventional and e-cigarettes promote atherosclerosis, as evidenced by increased arterial thickness and inflammatory markers. The cardiovascular risks associated with e-cigarettes are comparable to those of conventional cigarettes, highlighting the need for stricter regulation and public awareness.

## Background

Smoking remains a global public health concern, with over 1.2 billion smokers reported worldwide in 2021. Indonesia ranks third in smoking prevalence, with 69.1 million smokers, while electronic cigarette (e-cigarettes) use has risen sharply, reaching a prevalence of 3% by 2023 [1]. The popularity of e-cigarettes has surged due to aggressive marketing strategies that promote them as a safer alternative, their perceived effectiveness in smoking cessation, and the availability of various flavors that appeal particularly to young users. Despite being marketed as a safer option, e-cigarettes are increasingly popular among youth, even though evidence suggests they pose significant health risks. One such risk is electronic cigarette or vaping-associated lung injury (EVALI), with 889 cases reported by the CDC in 2020 [2].

Conventional cigarettes refer to traditional tobacco-based products that combust dried tobacco leaves, producing smoke containing thousands of harmful chemicals, including nicotine, tar, and carbon monoxide. In contrast, e-cigarettes are battery-operated devices that heat a liquid solution (commonly containing nicotine, propylene glycol, glycerin, and flavoring agents) to generate an aerosol, which users inhale. Both conventional and electronic cigarettes impair cardiovascular health through shared mechanisms, which include the disruption of nitric oxide (NO) production by nicotine, promotion of oxidative stress, and acceleration of atherosclerosis, hypertension, and cance [3]. Although marketed as safer, e-cigarettes emit toxic compounds such as aldehydes, volatile organic compounds, and heavy metals, which contribute to oxidative stress, endothelial injury, and vascular damage—similar to conventional cigarettes. Reactive oxygen species (ROS) further accelerate atherosclerotic plaque formation, underscoring their cardiovascular risks. While they may contain fewer harmful substances, e-cigarettes are not risk-free, warranting caution regarding their long-term effects [4, 5].

Atherosclerosis pathogenesis begins with endothelial damage, allowing low-density lipoprotein (LDL) infiltration into arterial walls, triggering inflammation and plaque development. Nicotine exposure from either cigarette type disrupts endothelial function, induces vascular inflammation, and accelerates histological damage, including elastic lamina fragmentation and reduced NO bioavailability [6, 7]. Given the rising prevalence of e-cigarettes use and its cardiovascular implications, this study aims to investigate the impact of conventional and e-cigarettes exposure on the development of atherosclerosis in Wistar rats (*Rattus norvegicus*), a widely used model for vascular research due to its similarity to human vascular responses [8]. Despite growing concerns, data on e-cigarette-induced vascular damage remain limited. This study addresses this gap by comparing endothelial dysfunction, oxidative stress, and atherosclerotic progression between e-cigarettes and conventional smoking. The findings aim to deepen understanding of cigarette-induced vascular aging and inform public health interventions.

## Methods

### Ethical statement

All experimental procedures were approved by the Animal Care and Use Committee (ACUC) of the Faculty of Veterinary Medicine, Universitas Airlangga (Protocol number 2.KEH.002.01.2024) and conducted in accordance with Indonesian animal protection laws [9]. Additionally, this study adhered to the ARRIVE guidelines for animal studies, enhancing reproducibility and transparency [10].

### Animals

Twenty-one male Wistar rats (*Rattus norvegicus*), aged 6–8 weeks and weighing 150–200 g, were obtained from the Laboratory Animal Center of Universitas Airlangga. Rats were based on normal activity levels, smooth fur, and the absence of anatomical abnormalities. Rats with signs of preexisting illness or abnormal grooming were excluded. Rats were housed in modified cages made of sturdy wood and mica (75 × 90 × 60 cm) with ventilation holes and smaller cages (25 × 17 × 12.5 cm) for individual exposure. The cages were equipped with air exposure apparatus, cigarette exposure devices, and proper ventilation. Cage ventilation was specifically designed to maintain carbon monoxide (CO) and CO₂ levels below toxic thresholds during exposure sessions, ensuring the safety of the animals.

The rats were acclimatized for seven days in group cages with food and water provided ad libitum, and housed in a controlled environment with a temperature of 20–24 °C, a 12-h light–dark cycle. Environmental enrichment, including nesting material and chewable objects, was provided to reduce stress. Noise and pollutants were minimized to avoid external confounders. To reduce experimental bias, rats were randomly assigned to groups using a computer-generated randomization sequence, and cage position and exposure chamber order were also randomized.

### Experimental design and cigarette smoke exposure

A randomized controlled experimental study was conducted using male Wistar rats to assess the effects of conventional and electronic cigarette exposure on atherosclerosis development. Sample size was determined using the Higgins and Kleinbaum formula, resulting in seven rats per group [11]. Rats were randomly divided into three groups: (1) a control group (no smoke exposure, exposed to smoke-free air); (2) a group exposed to conventional cigarette smoke; and (3) a group exposed to electronic cigarette smoke.

Both exposure groups were subjected to smoke inhalation for 30 min per day, 5 days a week, over 12 weeks. For conventional cigarette exposure, rats were exposed to side-stream smoke from commercially available Surya (Gudang Garam) cigarettes containing 2.2 mg nicotine per stick. A total of five cigarettes were used per exposure session. Smoke was pumped into the exposure chamber using a precision smoke delivery system, delivering 150 mL of smoke every 3 s to simulate human smoking exposure levels. The chamber was equipped with pollutant sensors to monitor carbon monoxide (CO) and CO₂ levels, ensuring safe exposure conditions. For electronic cigarette exposure, rats were exposed to vapor from FOOM e-cigarettes liquid containing 3% nicotine. The liquid was vaporized using a temperature-controlled vaporizer to simulate human vaping conditions. The exposure method was similar to the conventional group, with vapor pumped into the chamber at the same rate and volume. Efforts were made to standardize the nicotine dose in e-cigarettes to match that of conventional cigarette exposure, accounting for interspecies differences. This resulted in a dose of 0.37 mL/day for rats, administered during the 30-min exposure sessions, 5 days a week for 12 weeks.

### Blood sampling and tissue collection

The primary outcome of the study was intima-media thickness (IMT), a well-established marker of atherosclerosis. Secondary outcomes included the plasma concentrations of tumor necrosis factor-alpha (TNF-α) and monocyte chemoattractant protein-1 (MCP-1), which were measured as indicators of inflammation. At the end of the 12-week exposure period, the rats were anesthetized with ketamine (75 mg/kg) and xylazine (10 mg/kg) to ensure minimal discomfort during sample collection. Blood samples averaging 6–7 mL per rat were obtained via intracardiac puncture and carefully divided into EDTA-coated and heparin-coated tubes for subsequent plasma separation. Plasma samples were analyzed for TNF-α and MCP-1 concentrations using enzyme-linked immunosorbent assay (ELISA) with the *Elabscience* kit. All assays were conducted in duplicate to enhance reliability and accuracy, strictly following the manufacturer’s guidelines.

To collect the aorta and common iliac arteries, the thoracic and abdominal cavities of the rats were opened. The tissues were thoroughly washed with saline, fixed in 4% formalin, and prepared for histopathological examination. The collection of the aortic root adhered to the protocols outlined by Centa et al. (2019) to ensure consistency with established standards [12].

The collected aortic and iliac artery tissues were embedded in paraffin and sectioned to a thickness of 4 µm. These sections were stained with hematoxylin and eosin (H&E) and examined under a light microscope at 40 × magnification. Measurements of IMT were performed at four specific positions (12, 3, 6, and 9 o'clock) on the cross sections. Atherosclerotic lesions were classified based on the detailed criteria provided by Libby et al. (2011) [13]. To prevent bias, outcome assessors and data analysts were blinded to group allocations. Coding of samples was implemented throughout the study to maintain allocation concealment and ensure the integrity of the data analysis process.

### Statistical analysis

All data were analyzed using SPSS software (version 25.0 for Windows). Normality was assessed with Shapiro–Wilk tests, and nonparametric Kruskal–Wallis tests were used where assumptions were violated. One-way ANOVA, followed by LSD post hoc tests, was used to compare TNF-α, MCP-1, and IMT values across groups. Fisher’s test was applied to evaluate atherosclerosis incidence across groups, with p < 0.05 considered statistically significant.

## Results

### TNF-α concentration analysis

**Fig. 1:**
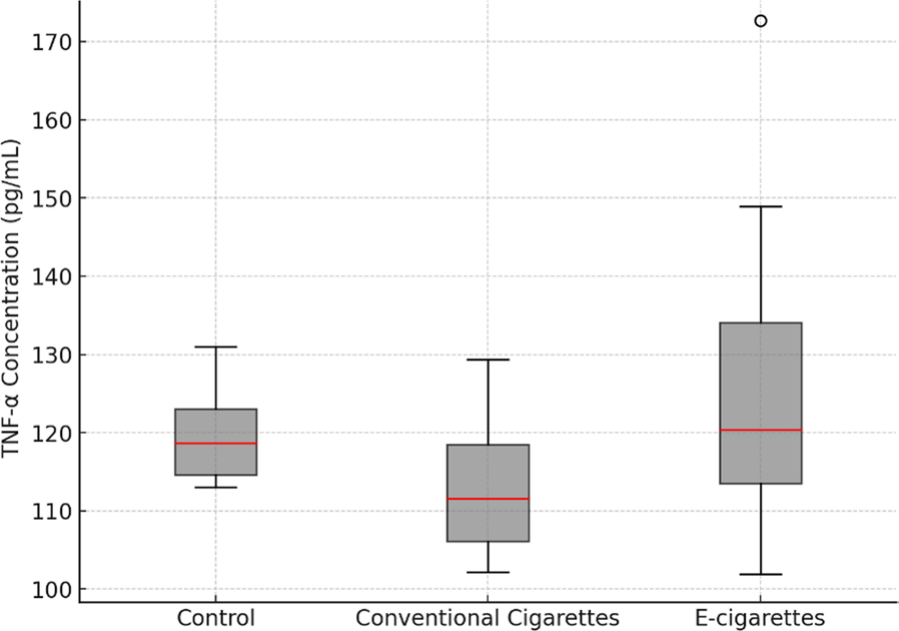
Changes in TNF- α levels across control and exposure groups

The mean TNF-α levels increased in both the conventional cigarette (117.57 ± 8.08 pg/mL) and electronic cigarette groups (130.00 ± 23.04 pg/mL) compared to the control group (117.14 ± 8.75 pg/mL). Despite this increase, the difference was not statistically significant (*p* = 0.217), suggesting that cigarette exposure may not substantially influence TNF-α levels under these experimental conditions. The changes in TNF-α levels across the control and exposure groups are illustrated in Fig. 1.

### MCP-1 concentration analysis

**Fig. 2:**
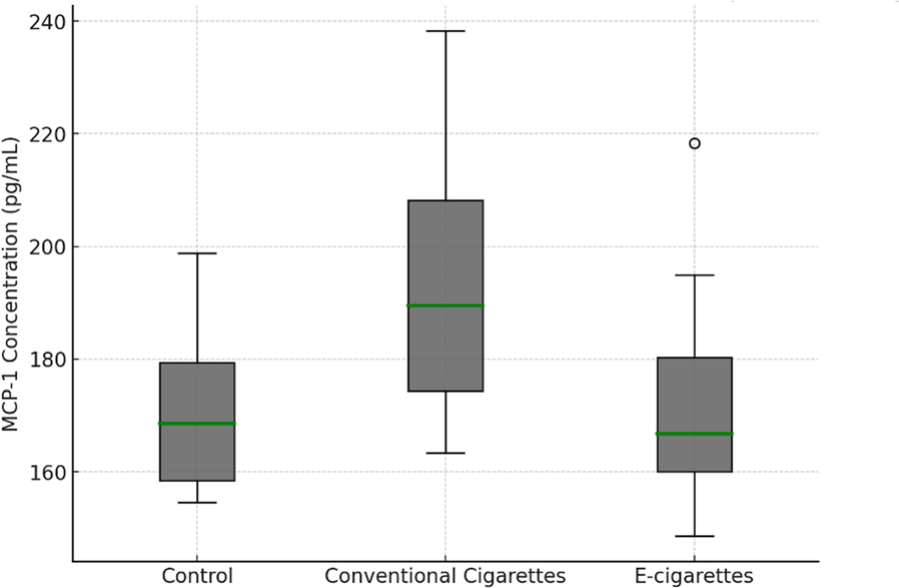
Changes in MCP-1 levels across control and exposure groups

MCP-1 levels were significantly higher in the conventional cigarette group (205.77 ± 22.18 pg/mL) compared to the control group (164.77 ± 21.57 pg/mL; *p* = 0.003). This indicates that conventional cigarette smoke strongly stimulates MCP-1 production, a marker of inflammation. The electronic cigarette group (176.28 ± 22.68 pg/mL) showed an increase compared to control, but this was not statistically significant (*p* = 0.344). Interestingly, a significant difference was observed between the conventional cigarette and electronic cigarette groups (*p* = 0.023), highlighting a stronger inflammatory response from conventional cigarette exposure. The changes in MCP-1 levels across the control and exposure groups are illustrated in Fig. 2.

### Histopathological examination of aorta and iliac artery

Microscopic examination revealed no atherosclerotic lesions in the control group. However, both the conventional and electronic cigarette groups exhibited distinct atherosclerotic changes in the aorta and iliac arteries. The lesions were characterized by fatty streaks, thickened intima-media layers, and disrupted elastic fibers, hallmarks of early atherosclerosis. A significant increase in atherosclerosis incidence was observed in the conventional cigarette group (*p* = 0.005) and the electronic cigarette group (*p* = 0.001) compared to the control group. No significant difference in lesion incidence was found between the two exposure groups (*p* = 1.00). The mean IMT of the iliac arteries increased significantly in both the conventional cigarette (90.49 ± 25.02 µm) and electronic cigarette groups (90.68 ± 12.26 µm) compared to control (68.50 ± 5.6 µm; *p* = 0.031) (Fig. 1). This finding reinforces the role of both cigarette types in promoting vascular remodeling. No significant difference was found between the two exposure groups (*p* = 0.983).

IMT measurements in the aorta revealed similar trends, with notable thickening in both the conventional cigarette (100.89 ± 25.60 µm) and electronic cigarette (83.75 ± 7.45 µm) groups compared to control (67.22 ± 3.07 µm). Analysis using the post hoc ANOVA test showed statistically significant differences between the control group and the cigarette exposure group (*p* = 0.03) as well as between the control group and the e-cigarette exposure group (*p* = 0.002). However, there was no statistically significant difference between the cigarette and e-cigarette groups themselves (*p* = 0.27). This observation aligns with earlier histological findings and supports the hypothesis that both cigarette types induce early vascular changes. IMT thickening in the aorta and iliac arteries was further validated through quantitative measurements and visualized in histological sections. Detailed IMT measurements for the aorta are presented in Table 1.

**Table 1:** Intima-media thickness (IMT) measurement (µm) in aorta

No	Control (μm)	Conventional cigarette (μm)	E-cigarette (μm)
1	66.5	89.75	77.75
2	61.25	102.75	86.5
3	68.5	75.25	90.5
4	69.9	86.5	75.75
5	68.75	84.5	88.5
6	65.75	117.75	92.5
7	69.9	149.75	74.75

**Fig. 3:**
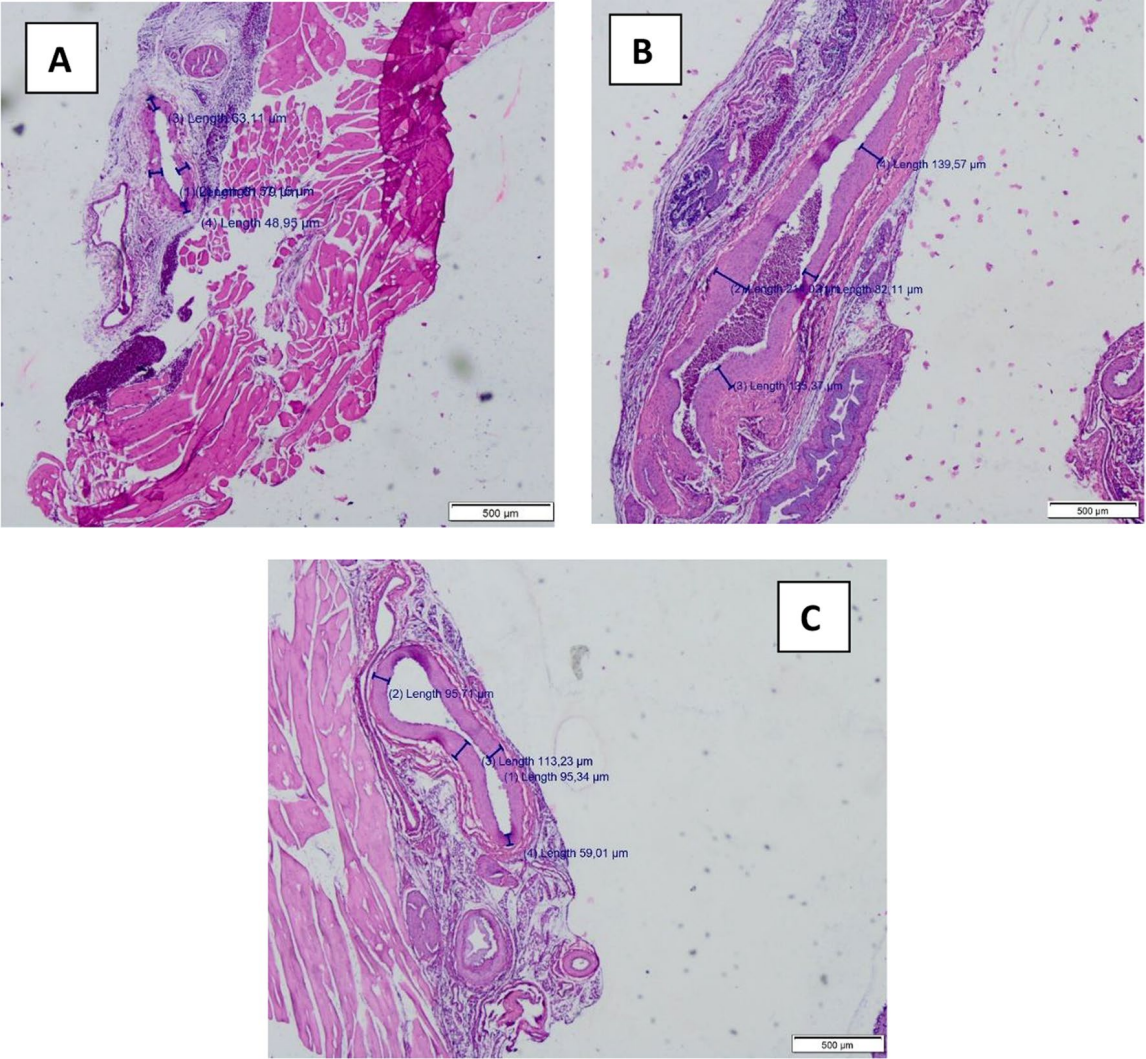
Microscopic examination of IMT of the iliac arterial wall tissues; **A** Control group; **B** Conventional cigarette group; and **C** E-cigarette group

**Fig. 4:**
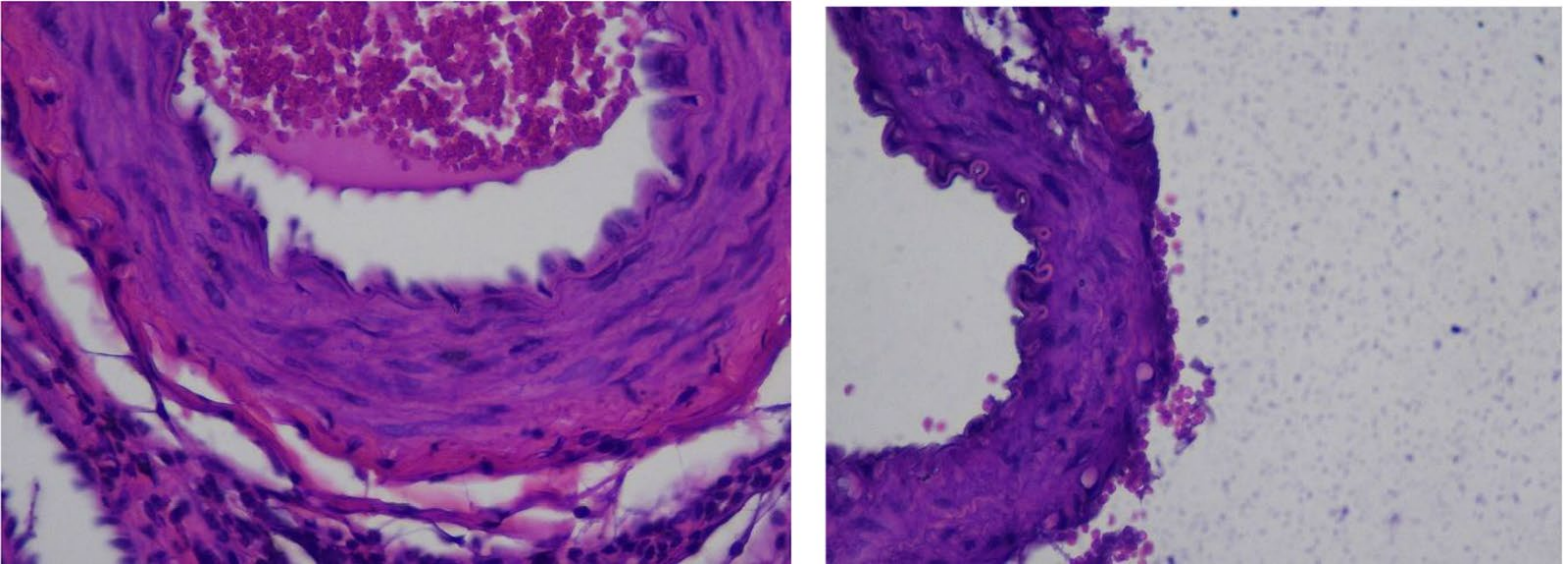
Microscopic examination of atherosclerosis lesion

Correlation analysis revealed a positive and significant correlation between IMT and atherosclerosis incidence (Pearson correlation coefficient = 0.651; *p* = 0.001). However, no significant correlations were found between TNF-α or MCP-1 levels and atherosclerosis incidence. Figure 3A shows an early stage atherosclerotic lesion, characterized by intimal thickening, fatty streaks formed by foam cells, and mild fiber degradation in the tunica media, with scattered inflammatory cells indicating an immune response. Figure 3B depicts a more advanced atherosclerotic lesion with pronounced fatty streaks, significant fiber degradation in the tunica media, elastic fiber fragmentation, and intimal thickening, leading to lumen narrowing and potential endothelial dysfunction (Fig. 4).

## Discussion

The results of this study demonstrated that both conventional and electronic cigarettes induce atherosclerosis in *Rattus norvegicus*. Significant increases in MCP-1 concentrations and IMT were observed in both smoke-exposed groups, confirming their pro-inflammatory and atherogenic potential. The findings indicate that both types of cigarette exposure pose comparable risks for the development of atherosclerosis. Although e-cigarettes are marketed as safer alternatives, their effects on the progression of atherosclerosis were similar to conventional cigarettes, indicating potential long-term cardiovascular risks.

### Molecular pathways and pathophysiology of atherosclerosis

Atherosclerosis is a multifactorial and progressive disease driven by chronic inflammation and oxidative stress, processes that are significantly exacerbated by exposure to both conventional and electronic cigarette smoke. The pathogenesis of atherosclerosis in both cases begins with endothelial dysfunction [14]. This dysfunction is triggered by the reactive oxygen species (ROS) and toxic compounds present in cigarette smoke, which impair the endothelial cells’ ability to produce nitric oxide (NO), a crucial molecule for vascular homeostasis and vasodilation [15]. The reduction in NO results in vasoconstriction and the establishment of a pro-inflammatory environment, predisposing the arterial walls to atherogenesis.

The impaired endothelium becomes more permeable to low-density lipoprotein (LDL) particles, which infiltrate the intima of the arterial wall and undergo oxidative modification to form oxidized LDL (oxLDL). OxLDL is a key player in atherosclerosis, as it is highly immunogenic and stimulates endothelial cells to express adhesion molecules like vascular cell adhesion molecule-1 (VCAM-1), facilitating the adhesion and transmigration of monocytes into the intima [12]**.** MCP-1 plays a crucial role in this process by recruiting monocytes to the site of endothelial dysfunction. Once inside the intima, these monocytes differentiate into macrophages and ingest oxLDL, transforming into lipid-laden foam cells. These foam cells, hallmarks of early atherosclerotic lesions known as fatty streaks, release pro-inflammatory cytokines such as tumor necrosis factor-alpha (TNF-α), further perpetuating inflammation within the arterial wall [13].

In this study, both conventional and electronic cigarette exposure led to increased levels of TNF-α and MCP-1, although the increase in TNF-α was not statistically significant. TNF-α is a potent mediator of inflammation and plays a critical role in the early stages of atherogenesis by activating nuclear factor kappa B (NF-κB), a transcription factor that upregulates the expression of inflammatory genes, including MCP-1 [16]. This suggests that TNF-α acts as an early trigger for the upregulation of MCP-1, which persists longer during the inflammatory response. The TNF-α-TNFR2 pathway, specifically, has been shown to increase MCP-1 levels by activating NF-κB in immune cells, contributing to chronic inflammation and plaque formation [17, 18].

Differences in MCP-1 levels between conventional and electronic cigarettes can be attributed to the distinct chemical compositions of these products. Conventional cigarettes, through combustion, generate numerous toxic compounds, including nicotine, tar, carbon monoxide, and aldehydes, which exacerbate inflammatory responses and elevate MCP-1. In contrast, electronic cigarettes lack combustion, which reduces the formation of harmful by-products like acrolein, a known contributor to atherosclerosis [17, 19]. However, despite producing fewer toxic compounds, electronic cigarettes still release irritants such as propylene glycol, glycerol, and nicotine, which contribute to inflammation and endothelial dysfunction, though potentially to a lesser extent than conventional cigarettes [20].

Cigarette smoke activates multiple molecular pathways that promote atherosclerosis. ROS generated by cigarette smoke can activate NF-κB and other transcription factors, leading to MCP-1 transcription. Additionally, the MAPK signaling pathway, including ERK, JNK, and p38 MAPK, is activated by smoke exposure and further drives MCP-1 production [21, 22]. These pathways contribute to endothelial dysfunction, a precursor to atherosclerosis, and reinforce the link between cigarette-induced inflammation and cardiovascular disease [23].

This study observed that both conventional and electronic cigarette exposures resulted in significant increases in IMT, a reliable marker of early atherosclerosis. The increase in IMT correlated with the incidence of atherosclerosis, consistent with studies showing a strong association between peripheral large artery atherosclerosis and the development of coronary artery disease [24, 25]. The iliac artery, used in this study, showed that smaller arteries are particularly susceptible to smoking-induced damage, further supporting the observation that chronic exposure to cigarette smoke contributes to early atherosclerotic changes in smaller vessels [26]. Overall, both conventional and electronic cigarettes contribute to the pathophysiology of atherosclerosis by activating key inflammatory pathways that lead to endothelial dysfunction, oxidative stress, and lipid accumulation. While electronic cigarettes produce fewer harmful combustion products, their long-term cardiovascular effects remain concerning due to their ability to trigger similar molecular pathways of atherosclerosis as conventional cigarettes [2, 27].

### Clinical implications and relevance

The clinical implications of these findings are profound, as they highlight the significant cardiovascular risks associated with both conventional and electronic cigarette use. Increased IMT, observed in this study, is a well-established marker of early atherosclerosis and is predictive of future cardiovascular events such as myocardial infarction and stroke [24]. Studies have shown that even a small increase in IMT correlates with a substantial increase in cardiovascular risk. IMT thickening in the iliac artery, as reported in this study, parallels findings from human studies linking elevated IMT to coronary artery disease and stroke [27]. This underlines the potential for both types of cigarette exposure to accelerate the development of atherosclerotic disease.

Fatty streaks, the earliest visible signs of atherosclerosis, were also noted in the study. These lesions, composed primarily of foam cells, can progress to more advanced forms of atherosclerosis if not addressed early. Their presence in young individuals is particularly concerning, as it suggests an early onset of atherosclerotic changes that could lead to significant cardiovascular morbidity later in life [28]. The findings from this study, indicating increased fatty streak formation in both smoke-exposed groups, suggest that electronic cigarettes may not be the safer alternative as they are often perceived to be [14].

These findings imply that electronic cigarettes, despite being marketed as safer alternatives, may not significantly reduce the risk of atherosclerosis [20]. The significant increase in IMT and fatty streak formation observed in this study implies that electronic cigarettes could pose similar long-term cardiovascular risks as conventional cigarettes, reinforcing the need for stricter regulation and awareness regarding their use. This has important implications for public health, as the use of electronic cigarettes could potentially lead to a new generation of individuals at risk for premature cardiovascular disease [29].

Translating these findings from animal models to human health requires careful consideration, but epidemiological studies have shown similar trends. For instance, smokers exhibit increased carotid IMT and a higher prevalence of atherosclerotic plaques compared to non-smokers. Recent studies have also reported that electronic cigarette users show signs of endothelial dysfunction and oxidative stress, similar to those observed in conventional cigarette users. This suggests that the harmful effects of electronic cigarettes may be underestimated. This study emphasizes the need for heightened awareness and regulation regarding both conventional and electronic cigarette use [21, 30, 31]. Despite being marketed as a safer alternative, electronic cigarettes activate similar molecular pathways of atherosclerosis as conventional cigarettes. Public health strategies should aim to reduce the use of both types of cigarettes to prevent the long-term cardiovascular complications associated with these products.

### Study strength and limitation

This study provides a valuable insight of the inflammatory and atherogenic effects of both conventional and electronic cigarette smoke in a controlled experimental setting using *Rattus norvegicus* as a model. By incorporating multiple biomarkers such as TNF-α and MCP-1 alongside IMT measurements, it provides a foundational understanding of the cardiovascular impact of these exposures**.** Despite its strengths, this study has several limitations. The use of animal models, while informative, may not fully replicate human responses to cigarette smoke exposure. Furthermore, the study duration of 12 weeks may not sufficiently capture the progressive nature of vascular injury and long-term cardiovascular consequences. Future studies should consider longer exposure periods, larger sample sizes, and multi-time-point assessments of inflammatory markers. Moreover, further investigation into additional molecular pathways, such as oxidative stress markers, endothelial dysfunction mediators (e.g., VCAM-1, ICAM-1), and cytokine signaling pathways, would help clarify the underlying mechanisms of cigarette smoke-induced vascular injury.

## Conclusion

This study challenges the perception of e-cigarettes as safer alternatives by highlighting their comparable cardiovascular risks to conventional cigarettes. The observed vascular changes suggest that e-cigarette aerosols contribute to endothelial dysfunction and early stage atherosclerosis. It indicates that e-cigarettes, often marketed as safer alternatives, pose comparable cardiovascular risks to conventional cigarettes. These findings underline the need for heightened public awareness and stricter regulatory measures regarding e-cigarettes use, particularly among youth and smokers seeking alternatives. Future research should explore the long-term cardiovascular effects of e-cigarettes, focusing on their role in vascular inflammation and oxidative stress. Integrating e-cigarettes into existing tobacco control policies is essential to mitigate their public health impact. Understanding their biological effects will be key to refining smoking cessation strategies and regulatory frameworks.

## Data Availability

No datasets were generated or analysed during the current study.

## Abbreviations

ACUC: Animal care and use committee
ARRIVE: Animal research: reporting of in vivo experiments
CDC: Centers for disease control and prevention
CO: Carbon monoxide
CO₂: Carbon dioxide
EVALI: Electronic cigarette or vaping-associated lung injury
ELISA: Enzyme-linked immunosorbent assay
H&E: Hematoxylin and eosin
IMT: Intima-media thickness
LDL: Low-density lipoprotein
MAPK: Mitogen-activated protein kinase
MCP-1: Monocyte chemoattractant protein-1
NF-κB: Nuclear factor kappa B
NO: Nitric oxide
oxLDL: Oxidized low-density lipoprotein
ROS: Reactive oxygen species
SPSS: Statistical package for the social sciences
TNF-α: Tumor necrosis factor-alpha
VCAM-1: Vascular cell adhesion molecule-1
